# Unveiling ophiuroid biodiversity across North Atlantic habitats via an integrative perspective

**DOI:** 10.1038/s41598-024-71178-9

**Published:** 2024-09-02

**Authors:** Lydia Anastasia Schmidt, Saskia Brix, Sven Rossel, Stefan Forster, Angelina Eichsteller

**Affiliations:** 1 Institute of Biological Science, University of Rostock, Albert-Einsteinstraße 3, 18059 Rostock, Germany; 2 Senckenberg am Meer, German Centre for Marine Biodiversity Research (DZMB), Martin−Luther−King−Platz 3, 20146 Hamburg, Germany; 3 Senckenberg am Meer, German Centre for Marine Biodiversity Research (DZMB), Südstrand 44, 26382 Wilhelmshaven, Germany

**Keywords:** Biodiversity, Biogeography, Genomics, Proteomics

## Abstract

The depths of the North Atlantic Ocean host a species-rich fauna providing heterogeneous habitats from thermal vent fields to cold-water coral reefs. With the increasing threat of destruction of deep-sea habitats due to human impacts, such as demersal fishing and the beginning of deep-sea mining, an analysis of the diversity and distribution of species is crucial for conservation efforts. Brittle stars occur in high biomasses, contributing to the biodiversity of the seafloor. Specimens were collected during several scientific expeditions to gain a more detailed insight into the brittle star diversity in the North Atlantic Ocean. An integrative approach to identify the species with DNA barcoding (mtCOI) in combination with morphological studies revealed 24 species. Most species have been previously identified in the North Atlantic, but sequences for 13 species are newly added to public repositories. Additionally, the MALDI-TOF-MS proteomic analysis was successfully applied for 197 specimens with known COI barcodes. Results are congruent with other molecular species delimitations demonstrating the functionality of proteomics for the identification of brittle stars. This dataset significantly expands our understanding of the taxonomic and genetic diversity of brittle stars and contributes to publicly available data. It emphasizes the importance of considering habitat heterogeneity for large scale patterns of biodiversity.

## Introduction

The North Atlantic Ocean (NA) is part of the Atlantic Ocean bounded by the equator in the south and the Arctic Ocean in the north^1,2^. The NA and the Arctic Ocean are interconnected through boundary currents that transfer heat into the Arctic^3^, where overturning surface water in the Labrador and Nordic Seas forms the North Atlantic Deep Water as part of the Atlantic meridional overturning circulation^4^. The bottom topography in the NA, especially around Iceland, is highly complex with processes such as geothermal activity along the central spreading axis creating hydrothermal vent fields along the Reykjanes Ridge or collapsed extinct spreading axis east of Iceland such as the canyon-like structure Aegir Ridge^5,6^. It provides the hard substrate for Vulnerable Marine Ecosystems (VME) indicator species like reef-building corals in the Lóndsjúp through the South of the Iceland-Faroe Ridge or sponge and crinoid gardens. Thus, the topography of the prominent ridges (Greenland-Iceland-Faroe Ridge, Reykjanes Ridge) create different habitats in the deep sea while interacting with the ocean circulation forming the structural composition of benthic communities^7^.

Since the end of the nineteenth century, more than 28 new habitats have been described showing the variation of the deep sea^8^. Abyssal plains, as the largest structure on earth, cover 75% of the ocean floor and are intersected by structures such as ridges, trenches, transform faults, and microplates forming unique environments characterized by different physical conditions^8^. Especially in shelf regions and at ridges, the habitat heterogeneity can be complemented by biotic structures formed by ecosystem engineers providing important habitats for benthic invertebrates and contributing to biodiversity^9^.

A major part of the benthic macro- and megafauna are brittle stars. Among echinoderms, the Ophiuroidea (brittle stars and basket stars) represent the most species-rich class, whose members are distributed worldwide from shallow waters to the abyssal zone^10^. Their reproductive strategies vary, ranging from asexual reproduction observed in some fissiparous brittle star species^11^ to brooding species that lack planktonic larvae^12^. However, most species are broadcast spawners, following a bipartite life cycle involving planktonic (feeding or lecithotrophic) larvae, which plays a central role in their potential distribution in marine systems, and a relatively immobile adult^10,13^.

Estimating biodiversity relies on the identification of specimens ideally at species level^14^. Especially for damaged specimens, cryptic species, or post-larval specimens lacking conspicuous characteristics, morphological identification is time-consuming and requires a high level of taxonomic knowledge^15^. Therefore, molecular approaches like DNA barcoding, using a species-specific molecular marker are an efficient tool for species identification of brittle stars^16–18^. Brittle stars serve as excellent organisms for biogeographical studies as they are often found in high densities and are recognized as dominant components in many benthic communities^19^.

Given increasing anthropogenic impacts on the benthic deep-sea environment, understanding patterns of biodiversity is crucial to implement conservation strategies^20,21^. Therefore, baseline studies are necessary to support public repository development, especially for the deep sea, where many species are still undiscovered and their impact within the ecosystem is not understood^18^.

As an alternative to DNA Barcoding, proteomic fingerprinting has been proven as a quick and less expensive method for the identification of marine species^22,23^. The proteomic fingerprint is obtained using matrix-assisted laser desorption/ionization–time of flight (MALDI-TOF) mass spectrometry and the resulting spectra can be used to identify species based on reference spectra^23^.

In this study the primary objective was to discover the deep-sea biodiversity of ophiuroids according to their habitats in the North Atlantic using an integrative approach by combining: I. morphological identification II. DNA barcoding and III. testing the capabilities of MALDI-TOF MS for species identification of these organisms.

## Material and methods

### Sampling and taxonomic determination

**Fig. 1:**
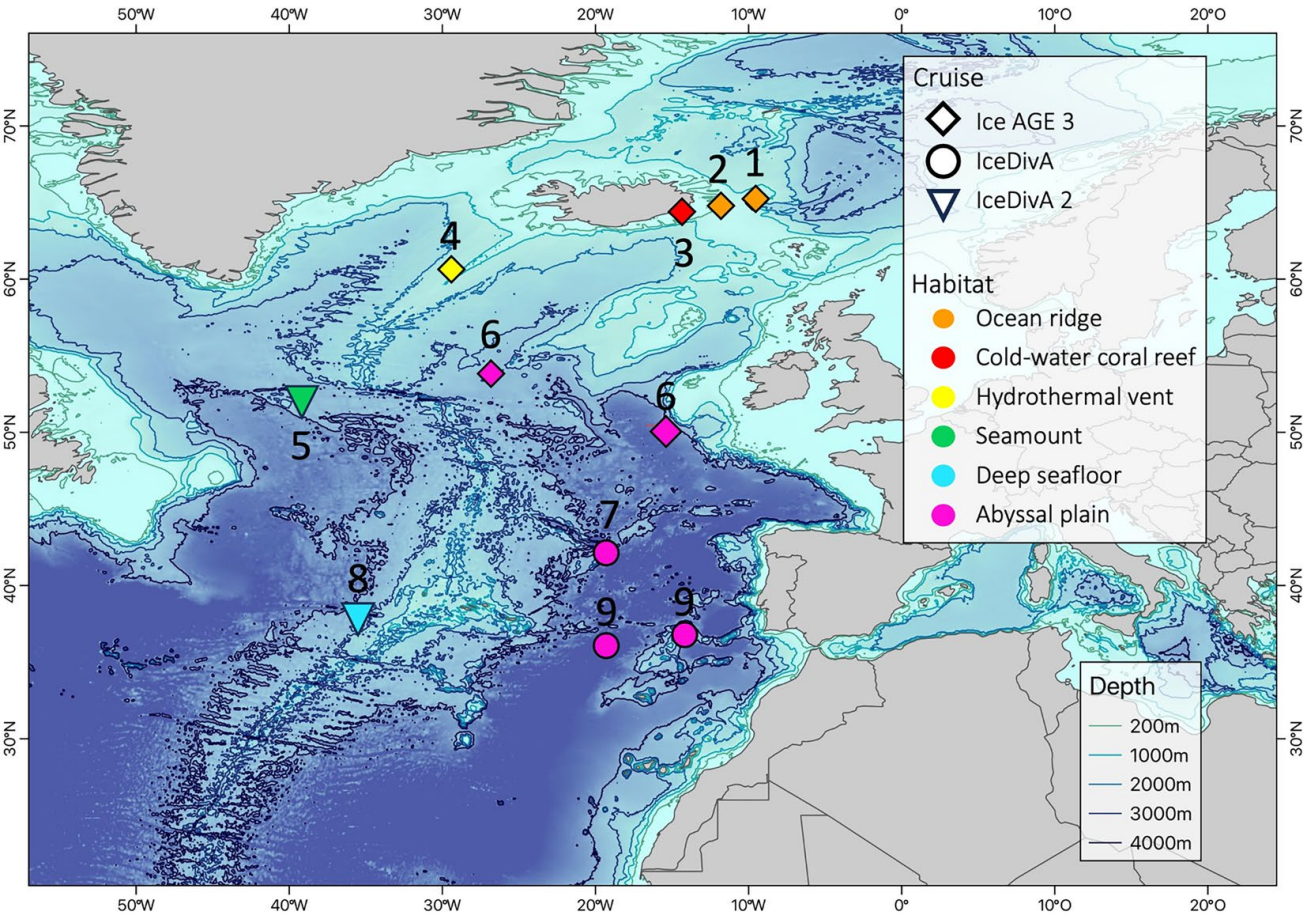
Bathymetric map of the sampling stations during the cruises IceDivA (SO280), IceDivA 2 (SO286), and IceAGE 3 (SO276). Each point represents one sampling area. The numbers represent the area where the stations are located (1) Aegir Ridge (2) Iceland-Faroe Ridge (3) Lóndsjúp (4) Vent field (5) NACES MPA (6) Abyssal plain 49N (7) Abyssal plain 41N (8) Azores south (9) Abyssal plain 36N. The symbol represents the cruise while the color indicates the sampled habitat. Map created in QGIS Version 3.32 (https://lima.qgis.org).

**Fig. 2:**
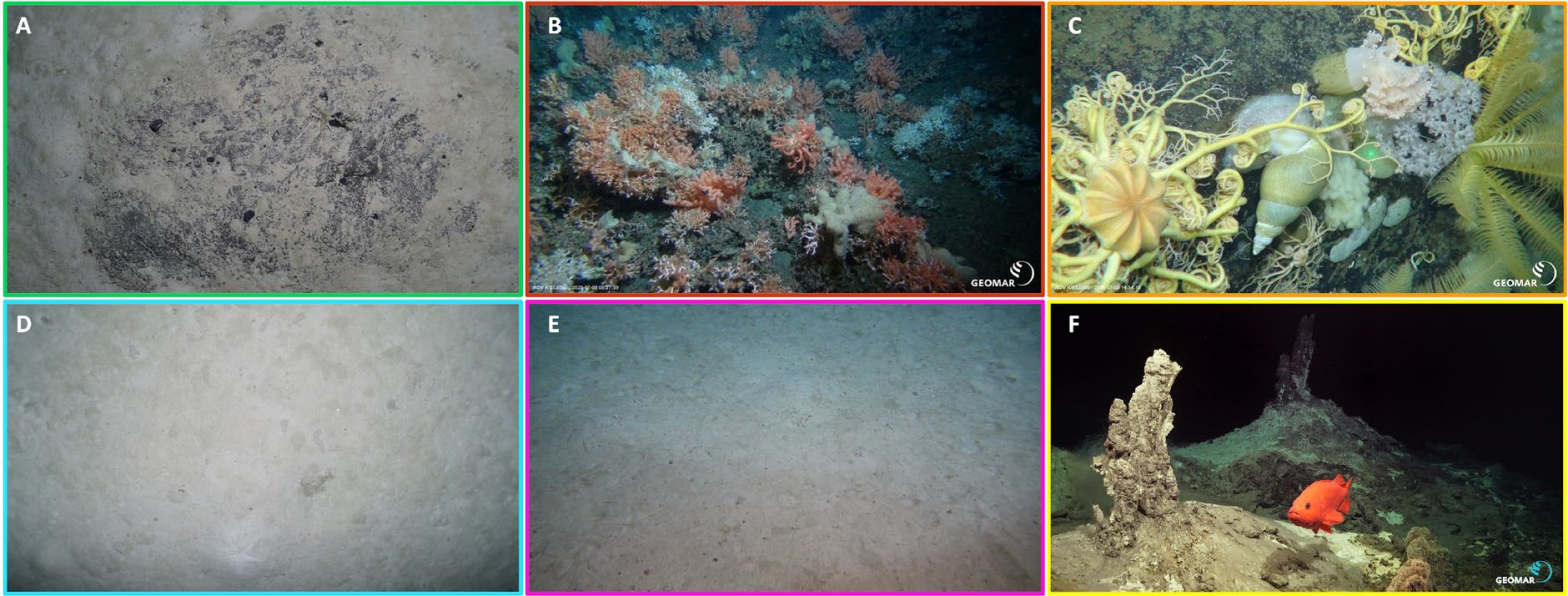
Exemplary illustration of the seabed characteristics within each habitat category: (**A**) Seamount base^25^ (**B**) Cold-water coral reef (SO276)^26^ (**C**) Ocean ridge (SO276)^26^ (**D**) Deep seafloor (SO286)^25^, (**E**) Abyssal plain (SO276)^26^. (**F**) Vent field (SO276)^26^.

Specimens were collected during several scientific cruises in the North Atlantic Ocean: IceDivA (SO280, 08.01.-07.02.2021)^24^, IceDivA 2 (SO286, 05.11.-08.12.2021)^25^, and IceAGE 3 (SO276, 22.06. – 26.07.2020)^26^. Samples from a total of 26 stations belonging to nine sampling areas were taken covering various habitats (Fig. 1). Furthermore, the habitats occurring in the sampling areas were classified following the definition by Ramirez-Llodra et al. (2010) (see Table 1) depending on their geomorphology (see Fig. 2).

**Table 1:** Sampling areas in this study.

Sampling area	Method	Latitude	Longitude	Depth (m)	Habitat	Description
Aegir Ridge	EBS/ ROV	64° 52.979′ N	009° 39.289′ W	1155	Ocean ridge	Steep slopes, hard substratum
Iceland-Faroe Ridge	EBS	64° 26.194′ N	011° 37.738′ W	416	Ocean ridge	Steep slopes, hard substratum
Lóndsjúp	ROV	64° 03.792′ N	014° 17.430′ W	407	Cold-water coral reef	Biotic structures, hart substratum
Vent field	ROV	60° 13.996′ N	029° 08.587′ W	661	Vent field, clear smoker	High temperature, unique mineral composition
NACES MPA	EBS	51° 57.600′ N	038° 59.375′ W	3677	Seamount base	Patches of hart substratum
Azores south	EBS	37° 13.551′ N	035° 32.276′ W	2973	Deep seafloor	Fracture zone, muddy sediment, small scale structured
Abyssal plain 36N	EBS	36° 02.328′ N 36° 28.794′ N	018° 59.497′ W 013° 59.471′ W	4380	Abyssal plain	Fine sediment
Abyssal plain 41N	EBS	41° 57.599′ N	018° 58.832′ W	4853	Abyssal plain	Fine sediment
Abyssal plain 49N	EBS/ ROV	49° 48.451′ N 53° 29.235′ N	015° 13.856′ W 026° 33.666′ W	4414	Abyssal plain	Fine sediment

The sampling method, average sampling depth and the associated habitat following Ramirez-Llodra et al. ^8^.

Samples were taken at depths ranging from 207 to 4904 m, covering a large geographical scale from 36 up to 64°N including diverse habitats comparing latitudinal transects on both sides of the MAR. The sampling was carried out using a Remotely Operated Vehicle (ROV Phoca or Kiel 6000, GEOMAR) and an Epibenthic sled (EBS) with a mesh size of 300 µm allowing the collection of small or post-larval ophiuroids. Specimens were transferred to 96% ethanol and stored at − 20 °C. All specimens were photographed after being preserved in ethanol using the Leica EC3 Camera. During morphological identification with a Leica M125 microscope, individual sample IDs were assigned to each specimen. For all specimens, except *Ophiocten* cf*. umbraticum* Lyman, 1878 which was identified using “Some Ophiuroidea from the Tasman Sea and adjacent waters”^27^, the morphological investigations are based on the taxonomic literature “Handbook of the echinoderms of the British Isles”^28^, “The taxonomy and zoography of the genus *Ophiocten* (Echinodermata: Ophiuroidea) in the North Atlantic Ocean”^29^, and “The deep-sea Ophiuroidea of the North Atlantic Ocean”^30^.

### Molecular analyses

DNA extractions were performed using 2–4 arm segments from the proximal part of the arm in 30 µl InstaGene™ Matrix (*Bio-Rad*) using the protocol^31^ or the E.Z.N.A.® TISSUE DNA KIT (*Omega Biotek*) according to the user manual. The extracted DNA was stored at − 20 °C at the German Centre for Marine Biodiversity Research (DZMB), Senckenberg am Meer, Wilhelmshaven. A 658 bp fragment of the mitochondrial cytochrome* c* oxidase subunit I (COI) gene was analyzed for DNA barcoding. The amplification of this fragment was performed in an Eppendorf AG Mastercycler using the echinoderm-specific forward primer I: LCOech1aF1 (5′-TTTTTTCTACTAAACACAAGGATATTGG-3′)^17^ and the universal reverse primer II: HCO2198 (5′-TAAACTTCAGGGTGACCAAAAAATCA-3′)^32^ following the protocol^33^. The PCR Mastermix (1 × 12.5 µl) consisted of 6.25 µl Accu Start PCR mix (2 × PCR master mix, Quantabio), 4.75 µl molecular grade H_2_O, 0.25 µl of each primer, and 1 µl DNA template. Negative controls were used in all runs.

Of the PCR products, 2 µl were run on a 1.5% agarose/TAE gel containing 1% GelRed to verify the amplification of a fragment with the correct size. PCR products were sent for sequencing to Macrogen (Amsterdam, Netherlands) after purifying with ExoSap-IT PCR (Product Clean-up Reagent, Thermo Fisher).

### Sequence analyses

All sequences were assembled, edited, and aligned using Geneious 7.0.2 (https://www.geneious.com). Sequences were matched using the NCBI “Basic Local Alignment Search Tool (BLAST)”^34^ against publicly available sequences for identification of post-larval specimens lacking meaningful characteristics for morphological identifications. Subsequently, the sequences were uploaded to the Barcode of Life Data System (BOLD, www. barcodinglife.org) project “DS-OPHNA Ophiuroids from the North Atlantic” (dx.doi.org/10.5883/DS-OPHNA). BOLD assigned these automatically to BINs which are considered comparable to species^35^.

The final alignment was subjected to phylogenetic analysis using Bayesian inference, performed in Beast2 version 2.7.5^36^ with a chain length of 10,000,000. The final alignment included selected sequences from GenBank, either from the species present in the dataset or from a sister species, to achieve better resolution for the Bayesian tree (Supplementary Table S1). The resulting ultrametric tree was analyzed using the Generalized Mixed Yule Coalescent (GMYC) method in R^37^ running the “splits” package^38^. The GMYC method classifies branches of a given tree based on likelihood values into intra- or inter-specific branching events, resulting in grouping putative species or other MOTUs together and supporting those with branch support values for statistical significance^39^.

Furthermore, we applied the Automatic Barcode Gap Discovery (ABGD) method. ABGD delimits the sequences into groups depending on genetic distances (p-distance), by calculating at which distance a so-called barcoding gap occurs in the distribution of pairwise genetic differences between the sequences of a given data set^40^. As a further source of delimitation, the BOLD BIN system was used^35^. Previously unknown sequences were assigned to a new BIN. The mean intra- and inter-specific genetic distances were computed using the Kimura 2-parameter model with 500 bootstrap replicates in MEGA v. 10.2.6^41^.

### Proteome measurement: MALDI-TOF MS

For proteomic fingerprinting, a tissue of the arm from adult specimens or whole post-larval specimens were used for MALDI-TOF MS measurements. The samples were incubated in ~ 10 µl α-cyano-4-hydroxycinnamic acid (HCCA) matrix solution. If initial results showed signs of improper preparation (according to^23^), the samples were diluted to obtain optimized mass spectra signals. After incubation for at least 5 min, 1.5 µl of the solution was transferred to a target plate and measured using a Microflex LT/SH System (Bruker Daltonics). Data was analyzed in R using R-packages MALDIquantForeign and MALDIquant^36^. Mass spectra were trimmed to an identical length from 2 to 20 kDa. Subsequently, spectra were square root transformed, smoothed using Savitzky Golay method^42^, baseline corrected using SNIP approach and normalized using total ion current method. Hierarchical clustering was carried out in R using Ward's D and Euclidean distances. Random Forest (RF)^43^ was done using the R-package randomForest, version 4.7.1.1^44^. Settings were used according to^45^ (ntree = 2000, mtry = 35, sampsize = number of specimens in the smallest class). T-distributed stochastic neighbor embedding (t-SNE) plots were created using R package Rtsne version 0.15^46^ with the following settings: perplexity = 20, max_iter = 4000 and theta = 0.

## Results

**Fig. 3:**
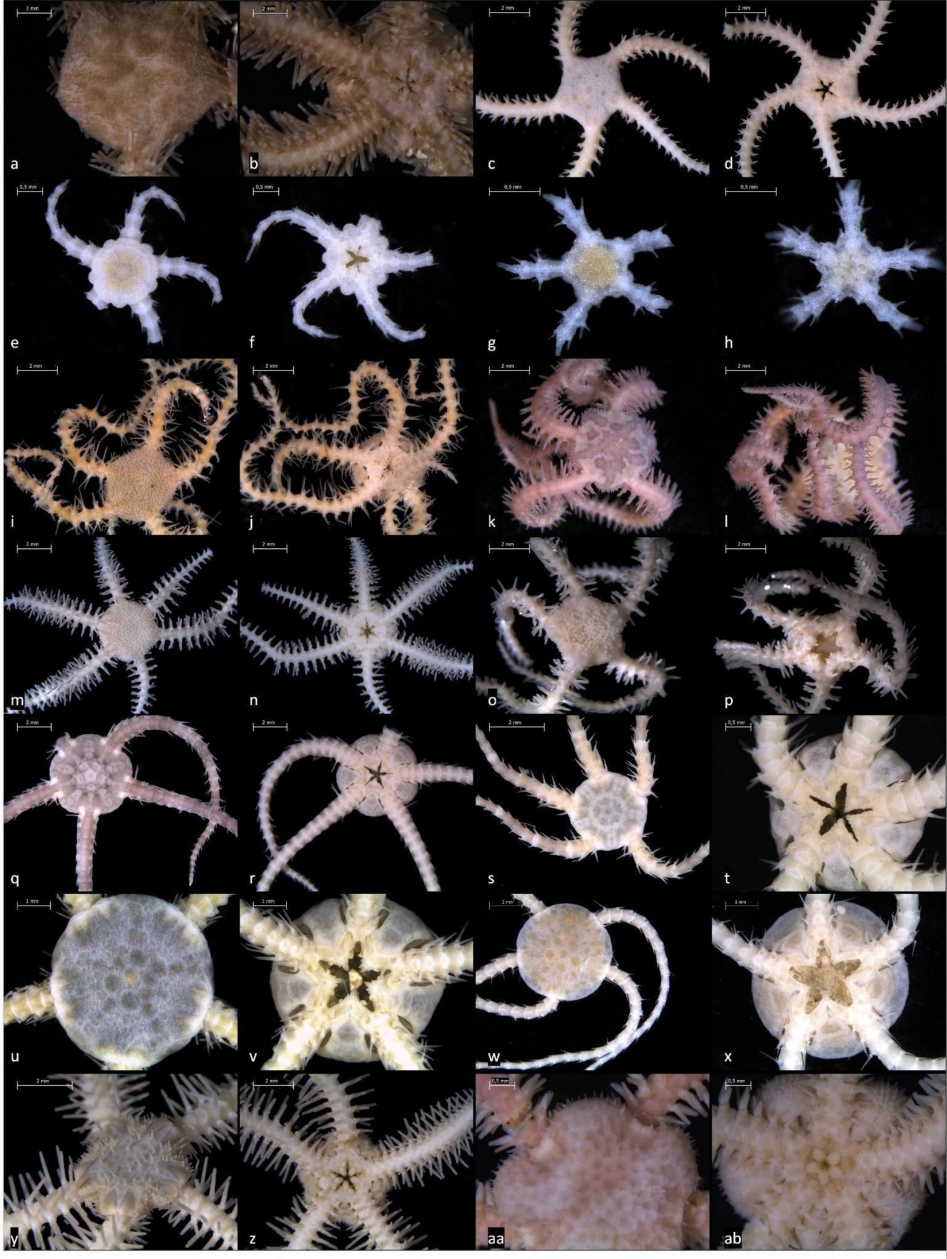
Selection of identified brittle star species. (**a**,**b**) *Ophiotreta spectabilis*, specimen 4661A: (**a**) dorsal view; (**b**) ventral view. (**c,d**) *Ophiopus arcticus*, specimen 2946: (**c**) dorsal view; (**d**) ventral view. (**e**,**f**) *Amphiophiura bullata*, specimen 8384B: (**e**) dorsal view; (**f**) ventral view. (**g**,**h**) post-larval cf. *Ophiuroglypha* A, specimen 6899A: (**g**) dorsal view; (**h**) ventral view. (**i**,**j**) *Ophiacantha bidentata*, specimen 5274: (**i**) dorsal view; (**j**) ventral view. (**k**,**l**) *Ophiopholis aculeata*, specimen 747: (**k**) dorsal view; (**l**) ventral view. (**m**,**n**) *Ophiosabine anomala*, specimen 3010: (**m**) dorsal view; (**n**) ventral view. (**o**,**p**) *Ophiosemnotes clavigera*, specimen 4121A: (**o**) dorsal view; (**p**) ventral view. (**q**,**r**) *Ophiura sarsii*, specimen 8758B: (**q**) dorsal view; (**r**) ventral view. (**s**,**t**) *Ophiura robusta*, specimen 1762E: (**s**) dorsal view; (**t**) ventral view. (**u**,**v**) *Ophiocten cf. umbraticum*, specimen 4286G: (**u**) dorsal view; (**v**) ventral view. (**w**,**x**) *Ophiocten gracilis*, specimen 620C: (**w**) dorsal view; (**x**) ventral view. (**y**,**z**) *Ophiactis abyssicola*, specimen 3011E: (**y**) dorsal view; (**z**) ventral view. (**aa**,**ab**) *Ophiactis balli*, specimen 8758H: (**aa**) dorsal view; (**ab**) ventral view. For each picture the individual scale is given.

In total, a 658 bp mtCOI fragment was successfully sequenced for 210 specimens. Among these, 162 specimens were identified morphologically as15 species. The remaining 48 specimens were either post-larval or juvenile, lacking sufficient morphological characteristics and thus remained morphologically unidentified. Using an integrative approach, a total of 24 species from 10 families (see Table 2) were detected. Of these, 23 species were confirmed by proteomic fingerprinting of 197 specimens using MALDI-TOF MS. For selected species, a morphotype is shown in Fig. 3.

**Table 2:** Overview of the species found in this study. One selected morphotype representing each species with the associated Specimen ID is shown.

Family	Species
Ophiacanthidae Ljungman, 1867	*Ophiacantha simulans* Koehler, 1895
	*Ophiacantha aculeata* Verrill, 1885
	*Ophiacantha bidentata* (Bruzelius, 1805*)*
	*Ophiacantha fraterna* Verrill, 1885
	*Ophiosemnotes clavigera* (Ljungman, 1865)
	*Ophiosabine anomala* (G.O. Sars, 1872)
Ophiohelidae Perrier, 1893	*Ophiotholia sp.*
Ophiosphalmidae O'Hara, Stöhr,	*Ophiosphalma armigerum* (Lyman, 1878)
Hugall, Thuy & Martynov, 2018	*cf. Ophiosphalma* sp.
Ophiopyrgidae Perrier, 1893	*Ophioplinthus* sp.
	*cf. Ophiuroglypha* sp. A
	*cf. Ophiuroglypha* sp. B
	*Amphiophiura bullata* (Thomson, 1877)
Ophiuridae Müller & Troschel, 1840	*Ophiura ljungmani* (Lyman, 1878)
	*Ophiura robusta* (Ayres, 1852)
	*Ophiura sarsii* Lütken, 1855
	*Ophiocten cf. umbraticum* Lyman, 1878
	*Ophiocten gracilis* (G.O. Sars, 1872)
Ophiotomidae Paterson, 1985	*Ophiotreta spectabilis* G.O. Sars, 1872
Ophiactidae Matsumoto, 1915	*Ophiactis balli* W. Thompson, 1840
	*Ophiactis abyssicola* M. Sars, 1861
Ophioscolecidae Lütken, 1869	*Ophiolycus purpureus* (Düben & Koren, 1846)
Ophiopholidae O'Hara, Stöhr, Hugall, Thuy & Martynov, 2018	*Ophiopholis aculeata* Linnaeus, 1767
Amphilepidida *incertae sedis* (temporary name)	*Ophiopus arcticus* Ljungman, 1867

### Species delimitation

The morphological identification of the 15 morphospecies was supported by molecular barcoding. Using different delimitation methods (GMYC, ABGD, and the BOLD BIN system) operational taxonomic units (MOTUs) were identified (see Fig. 3). Application of the GMYC model resulted in 27 MOTUs, the BIN system recognized 26 BINs, and ABGD resolved 25 MOTUs within the dataset.

All sequences were compared to the GenBank nucleotide database using BLAST. This comparison confirmed 11 species identifications that matched the morphological results. Nine MOTUs, which contained only post-larval specimens, could not be verified morphologically due to insufficient characters. However, they had BLAST matches at the genus level (*Ophiotholia sp*., *Ophioplinthus sp*.), with uncertainties for three of these MOTUs (cf. *Ophiuroglypha sp.* A, cf. *Ophiuroglypha sp*. B, cf. *Ophiosphalma sp*.) .

**Fig. 4:**
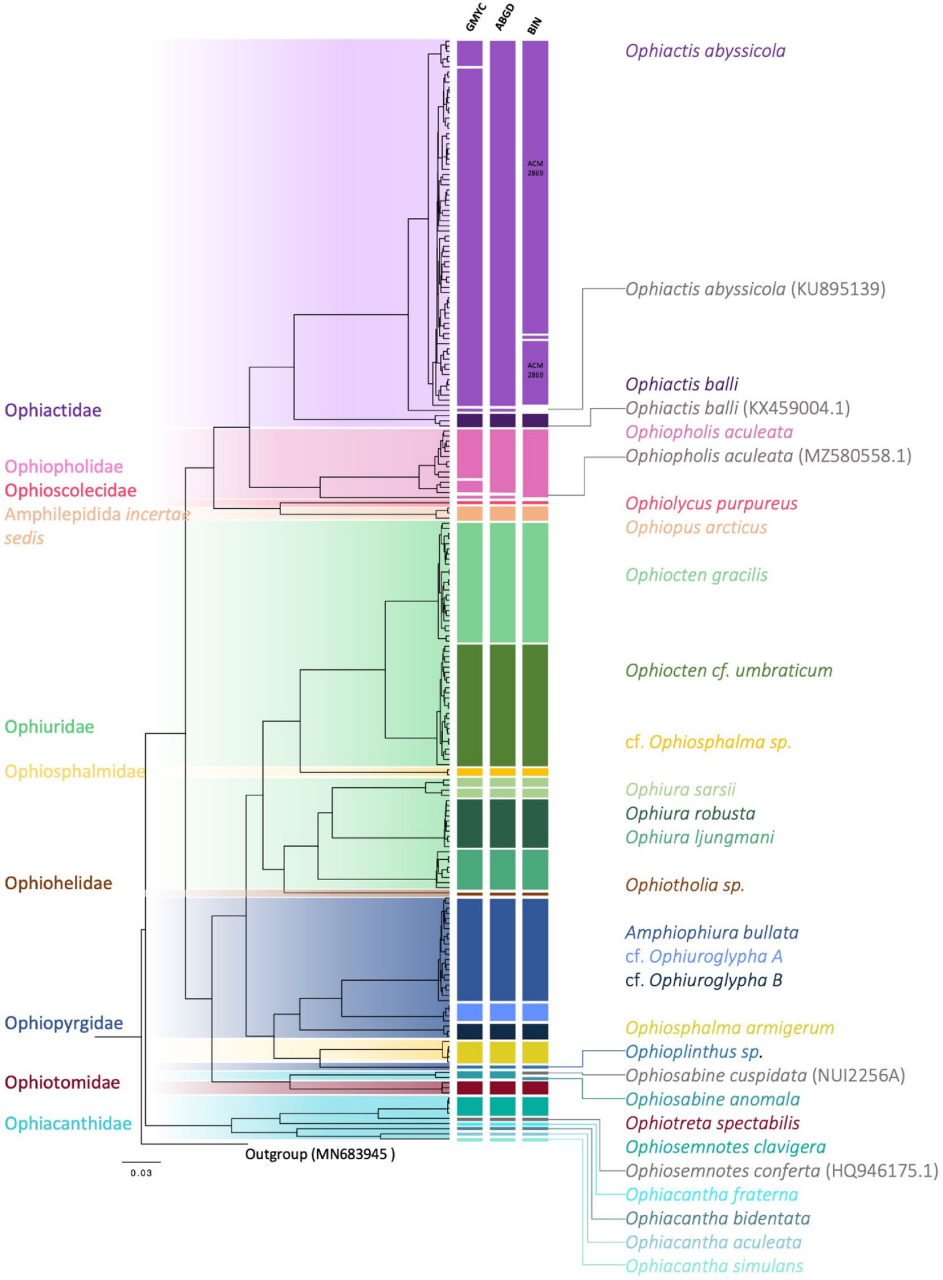
Ultrametric BI tree with the applied GMYC model of 219 mtCOI sequences from North Atlantic and Arctic brittle stars. The mtCOI fragment with a length of 658 bp and the additional GenBank sequences (see Supplementary Table S1) were computed with a strict clock model and an MCMC chain length of 10,000,000. The colored vertical bars represent the results of the applied species delimitation methods (GMYC, ABGD (prior maximal distance *P* = 0.0215) and the BIN system). The species to which the sequences are assigned is marked on the right with the associated families highlighted to the left. The sequences downloaded from GenBank are marked with a gray line.

From the 210 mtCOI sequences and additional sequences from GenBank (see Supplementary Table S2) an ultrametric tree was generated using Bayesian inference. To this tree, GMYC was applied (see Fig. 4) resulting in a total of 31 MOTUs including the GenBank sequences, the outgroup, and 27 MOTUS from our data. The BIN system distinguished between a total of 26 different BINs of which eight were new to the system. ABGD on the other hand resulted in a total of 25 different MOTUs when excluding GenBank sequences. The mean K2P distance within the MOTUs was 0.99%.

Slight differences were found between the results of the species delimitation models. The GMYC model split the *Ophiactis abyssicola* morpho group recovering two clades among the MOTUs from our own samples whereas the GenBank sequence (*O. abyssicola* from New Zealand (NZ), accession number: KU895139) clustered separately from these. ABGD recognized all sequences of *O. abyssicola* from our data set as the same MOTU while the NZ specimen forms a separate MOTU. Although the NZ sequence was not available to the BOLD system, it still found two different BINS for this species which were however not congruent to the ones found by the other methods. One BIN contained only specimen number ID2061 (BOLD: AFM5457) and one contained all remaining specimens from this morphotype (BOLD: ACM2869). A similar pattern was recovered for *Ophiopholis aculeata*. The GMYC model suggested two splits within the *Ophiopholis aculeata* morphogroup into three MOTUs, supported by 100% GMYC branch support. Two of these MOTUs consisted entirely of our specimens whereas the single sequence downloaded from GenBank (*Ophiopholis aculeata* from the Pacific, accession number:MZ580558.1) formed a distinct MOTU. The ABGD model, while agreeing on the split between our data and the GenBank sequence, did however not show the second split between the two clades from our data set. The BIN system assigned the same BIN (BOLD: AAA9003) to all specimens.

All molecular species delimitation methods split *Ophiura sarsii* into the same two clades (BOLD:ACO7183 and BOLD:AAD8851) which are morphologically indistinguishable.

Another notable distinction from this was *Ophiosabine anomala*. With the addition of another sequence downloaded from GenBank (*Ophiacantha cuspidata* from Ireland, accession number: NUI2256A), there is ambiguity for this species as ABGD and GMYC agreed that this sequence falls into one MOTU with *Ophiosabine anomala* from the dataset. However, the BOLD BIN system recognized our *Ophiosabine anomala* and the GenBank sequence of *Ophiosabine cuspidata* as two separate BINs with intra-MOTU genetic distances of 1.76% (p-dist). The remaining morphospecies were widely assigned to the same MOTUs by all three delimitation methods.

### MALDI-TOF-MS analysis

**Fig. 5:**
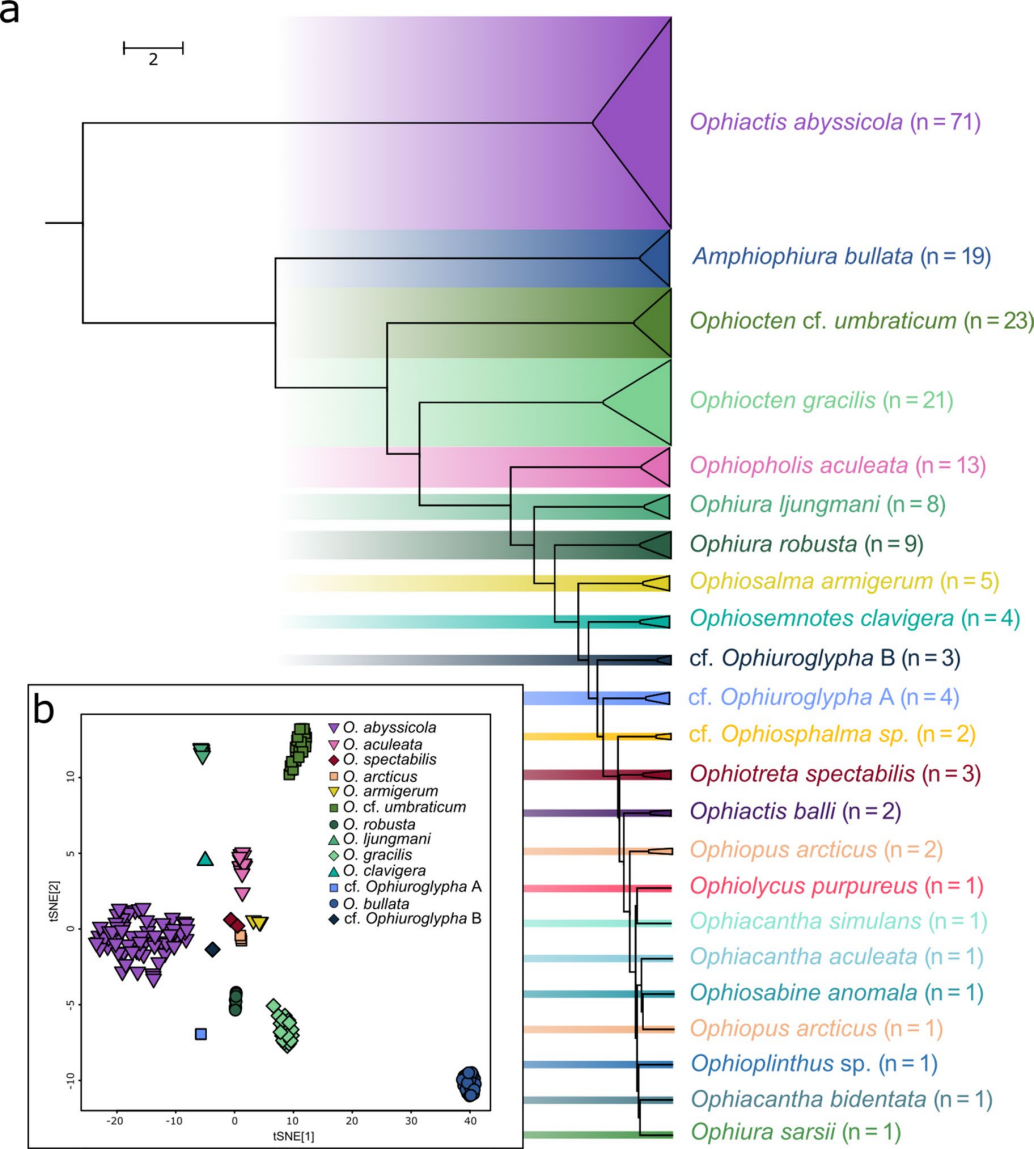
MALDI-TOF MS analyses. (**a**) Hierarchical clustering based on 197 protein mass spectra. Colors indicate species affiliations of collapsed clusters. Except for a single *O. arcticus* specimen, all specimens cluster according to their morphological and genetic identification. **(b)** TSNE plot resulting from a RF model including all species with at least three specimens. None of the specimens, including the single *O. arcticus*, was misclassified within the RF model indicating its usability in a classification approach.

Successful measurements were obtained from 197 specimens across 23 morphotypes. No mass spectrum was obtained from the single *Ophiacantha fraterna* specimen. Therefore, this species is not listed in the results for the proteomic analyses. The hierarchical clustering resulted in species-specific clusters, which are widely congruent to the morphological identification (see Fig. 5a). Only one *Ophiopus arcticus* specimen did not cluster alongside the remaining two specimens of the morphotype. However, the RF model, visualized in a tSNE plot (Fig. 5b) shows homogeneous grouping of all species, including the *Ophiopus arcticus* specimens, confirming the results of the molecular genetic and morphological analyses.

### Geographical distribution of the brittle star species

**Fig. 6:**
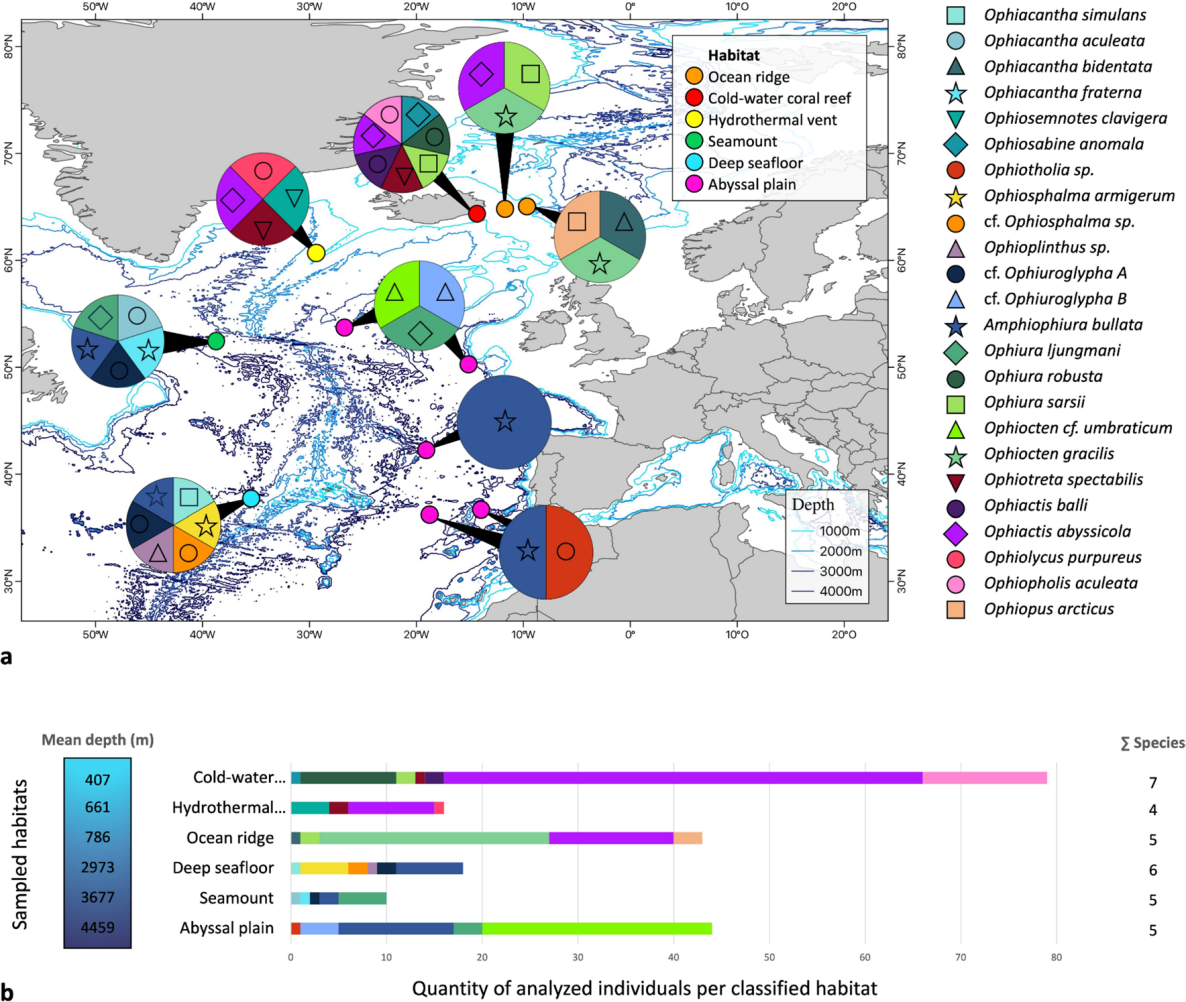
Distribution of the different species according to the various sampling areas and habitats, along with a visualization of the brittle star species composition in the classified habitats. Species are represented by specific colors and symbols, which are consistent across both subfigures for clarity. **(a**) The six habitats are indicated on the map with the associated color shown in the legend. The species occurring at a site are indicated in the circles associated with the respective sites by their color and symbol. Map created in QGIS Version 3.32. (**b**) For each habitat, the total number of barcoded specimens is shown, and the associated species is represented by the different colors of the bars. The total number of species is shown with gray numbers.

The species diversity by site is shown in Fig. 6a and ranged from one to seven species per sampling area. The species richness varied by habitat, ranging from four species in the hydrothermal vent field to seven species in the cold-water coral reef field. The number of specimens examined varies between 10 and 79 per habitat and is shown accordingly in the graph (see Fig. 6b). Based on the mean depth of the six different habitats, there was no recognizable diversity gradient concerning the depth.

## Discussion

### Differences in species delimitation models

By combining morphological diagnostics, the output of the molecular species delimitation methods and the application of proteome fingerprinting, we conclude to have collected and identified a total of 24 deep-sea ophiuroid species belonging to ten families. Of these 24 species, 11 had no molecular data available in public repositories. The specimens identified as *Ophiocten cf. umbracticum* could not be confidently identified to species level according to available keys, possibly constituting a new species.

Discrepancies were found between the methods used to delimit the species. Disagreements in delimitation between GMYC, ABGD, BIN and morphology occurred at splits within *Ophiactis abyssicola, Ophiopholis aculeata, Ophiura sarsii* and *Ophiosabine anomala.*

Splits within the *Ophiactis abyssicola* morphotype had been identified in previous studies, where sequences from the NA and Western Indian Ocean shared one clade, while sequences from the Southwest Pacific formed a separate one^47^. Thus, while there appear to be geographically patterned lineages within the species, these are not reflected in our data presumably because all our samples were obtained in the NA. However, both the ABGD and GMYC models showed consistent delimitation of our sequences and the NZ sequence. Because the respective sequence was not available in the BOLD system, this could not be supported by the BIN model. Consequently, this species either exhibits an enormous geographic range with surprisingly high intraspecific variability but without morphological variations, or they actually constitute distinct species^48^. Our data combined with previous findings may suggest the potential existence of a cryptic-species complex caused by geographical isolation that would demand further investigation^49^. Additionally, it is worth considering that *Ophiactis abyssicola* is recognized for its morphological variability^30^. Therefore, a comprehensive analysis of the morphology within this clade may further support the hypothesis of cryptic speciation.

*Ophiopolis aculeata* was split into three clades by both GMYC and ABGD while in the ABGD model only the downloaded sequence from a Pacific specimen forms a separated clade. All specimens from the NA were sampled at the Lóndsjúp and shared typical characteristic features of *Ophiopholis aculeata* such as smaller plates surrounding and separating the dorsal arm plates^30^. Since all sequences, including the downloaded sequence, share the same BIN number, the clades likely represent different mtCOI lineages.

*O. sarsii* showed a similar pattern in our study as was previously identified^50^, where two major mtDNA lineages were found in the Atlantic Ocean. Our results support this pattern as all species delimitation methods are consistent.

The splits within the *Ophiopholis aculeata, Ophiura sarsii* and *Ophiactis abyssicola* morphotypes might indicate cryptic lineages or ongoing speciation^49^ however it is also known that GMYC tends to overestimate species diversity^33,51^. Generally, the three molecular species determination methods reveal similar results, although GMYC tends to result in more clades than the ABGD model and the BIN system. While the GMYC and the ABGD model are based on the relative distances among sequences in the alignment, the BIN system compares uploaded sequences with available sequences in the BOLD database^38,40^.

It is noticeable that the BIN system was the only delimitation method that differentiated between *Ophiosabine cuspidata* (downloaded sequence) and *Ophiosabine anomala* (from this study). This special case reveals potential pitfalls of non-integrative identifications. Closely related species may quickly be misidentified or falsely assigned, highlighting the importance of a sufficiently large dataset and degree of sampling per species for reliable species discrimination^52^.

Generally, the results from proteomic fingerprinting aligned well with results from barcoding and morphological identifications. Only for a single species, *O. arcticus*, ambiguous clustering of protein mass spectra was found with one specimen clustering outside the main species-cluster. A visual comparison of the three spectra showed similar basic mass-spectrum patterns without any obvious quality differences that could be responsible for incorrect clustering. However, in a RF Model that could be used in future classification approaches, none of the *O. arcticus* specimens would be classified as another species from the data set, thus verifying its position within the other two specimens of the same species. As in other studies before, proteomic fingerprinting was proven to be a reliable and quick method for species identification applicable in biodiversity assessments^45,53,54^.

In this study, molecular species delimitations were combined with results from morphological identification and supported by proteome fingerprinting. This highlighted the importance of complementary interpretation of morphological and molecular tools, which has previously been identified as leading to better resolution of biodiversity^55,56^.

### Habitat heterogeneity and species richness

Various hypotheses attempt to explain the patterns of biodiversity and the “paradox” of high diversity in the deep sea^57^. One proposes that habitat heterogeneity, leading to a greater number of microhabitats, promotes higher biodiversity^9^. Variation of distinct geological structures and associated biotic and abiotic factors create unique niches that require species to adapt and thus drive species separation. In this study, different habitats were classified by their habitat structure and substrate availability and then used to derive the biodiversity patterns of brittle stars. Although different habitats occur across the NA, no notable differences in relative species diversity were recognized between the different habitats. However, there were clear differences in the species composition of the individual sampling regions.

The habitat with the highest recorded species richness (n = 7) was the cold-water coral (CWC) reef of Lóndsjúp located at the Aegir Ridge. This was the shallowest sampling station and closest to shore. The community is primarily composed of two coral species, *Desmophyllum pertusum* (Linnaeus, 1758) and *Madrepora oculata* Linnaeus, 1758^58,59^. In this study, *Ophiosabine anomala* (n = 1) and *Ophiura robusta* (n = 2) were the only species exclusively found at the Lóndsjúp, although neither one of them are known to be specifically associated with CWC as habitat^30,60^. The high species richness found at Lóndsjúp might be due to the 3D-structure provided through the corals enhancing the availability of microhabitats and function for the brittle stars depending on their species-specific biology^9^. Cavities between the corals can function as refuge and shelter, especially for juvenile and small brittle stars as documented for *Ophiopholis aculeata*^61^. While climbing on the coral, their elevated position may enhance foraging opportunities by providing access to additional food sources. *Ophiactis abyssicola*, found in high abundance at the Lóndsjúp, was observed perching on corals with their arms reaching into the water column, potentially reflecting an advantageous elevated position for particle capturing^16^.

Ocean ridges enhance the abundance of benthic organisms by extending and elevating the seafloor and providing hard substratum^20^. Thus, providing habitat for species typically found on continental margins^21^ that may typically not survive on abyssal plains^5,62^. Additionally, hard substratum can be used by other organisms (e.g. corals and sponges) to create biotic structures that are in turn used by brittle stars to extend their otherwise benthic position into the water column^9,63^. The ridge structures themselves can also provide cracks and caves that are used as shelter by brittle stars to be protected against predators^16^. Additionally, ridges can influence the environmental conditions^21^, for example, the Greenland-Iceland-Faroe Ridge creates different habitats in the northern Atlantic^64^. Resulting variations of abiotic factors can lead to variability in the species distribution^21^. The steep slopes also affect ocean currents redistributing food availability which influences the species richness and abundance on the deep-sea floor on a regional as well as on a larger geographical scale^65^. Even though the structure and sediment composition of the ocean ridges potentially offer various microhabitats, they can also act as functional distribution barriers inhibiting the cosmopolitan distribution of species^7^. The significance of potential barriers can vary between species depending on their life history.

Along ocean ridges, seamounts disrupt the abyssal plain and create a distinct habitat compared to the surrounding seafloor (8) with potentially altered food supply to the deeper ocean^21,66^. A special region in this dataset is the abyssal hill in the NACES MPA region, a component of the largest marine protected area in the North-East Atlantic^67^, situated at the base of the recently discovered seamount "Mount Doom"^25^. The sediment in this area is significantly influenced by the adjacent seamount, characterized by volcanic glass, which provides a hard substratum for benthic species.

The ophiuroid fauna at the investigated seamount overlaps with both the abyssal plain and the deep seafloor, as indicated by the presence of species such as *O. ljungmani* and *A. bullata* and cf. *Ophiuroglypha* sp. A (postlarval specimen). This is consistent with previous studies^68,69^ in which the ophiuroid fauna on seamounts reflects species found elsewhere at comparable depths and on the surrounding seabed. Despite the small sample size of ten specimens from this site, the identification of five different species shows a remarkable level of biodiversity in the seamount area. This observation is consistent with the findings of^68^ and may reflect the species richness associated with the highly variable topography, substrate, and bathymetry of seamounts at smaller scales^70^, which means that seamounts are not necessarily centers of endemism for brittle stars^69^.

Chemosynthetic ecosystems have been found at hydrothermal vents and cold seeps that have a primary production due to chemoautolithotrophic bacteria^8,71^. The hot, chemical-rich fluid that exists at hydrothermal vents can support reducing microbes creating a specific and unique habitat patch, with an associated community around the vent^71^. Even though chemosynthetic environments are known to host a variety of endemic species in various taxa^72^, including brittle stars^73^, none were discovered within this study. It is known that species from non-chemosynthetic habitats do visit vent sites and hydrothermal conditions^73^. All four species *Ophiactis abyssicola, Ophiotreta spectabilis, Ophiolycus purpureus* and *Ophiosemnotes clavigera* found at the vent field are often associated with cold-water corals and sponges^16,74,75^

Compared to the obvious variety of the habitats mentioned above, the fine sediment of the abyssal plains consists mainly of biological particles produced by planktonic organisms with siliceous or calcareous features and rock weathering on land^8^. This relatively homogeneous composition is interspersing habitats like abyssal hills or rocky habitats that provide heterogeneity^76,77^. This could lead to an underestimation of biodiversity due to the presence of more hard substratum habitats and small-scale differences in the abyssal terrain^77^. In this study, stations classified as abyssal plains showed no inhibited biodiversity. Although species richness did not differ from other habitats, the dominance of a few species like *Amphiophiura bullata* in individual sampling regions is remarkable.

Comparing the appearance, the deep seafloor area of the Azores has similar characteristics to the abyssal plains: fine sediment and limited hard substrate. However, this sampled area is in the broader region around the MAR, which is characterized by faults, fracture zones and abyssal plains covered with volcanic rocks^78^. Fracture zones showing high species diversity were observed on the steep slopes and rocks^79^. The geomorphological elements with steep vertical walls^25^ of the close-by fracture zone also influence seafloor diversity by potentially channeling ocean currents, increasing food availability and thus improving conditions for filter-feeders at the surrounding abyssal plains^21,80,81^.

In the deep sea, some species can exhibit a widespread distribution^48,82^ even if a uniform species distribution is exceptional^10,70^. The potential distribution range of individuals is a significant factor in structuring populations and the role of dispersal strategies for connectivity has been discussed^56,83^. Most benthic organisms have a bipartite life cycle with a planktonic larvae phase which plays a central role in the potential distribution in marine systems^56^. The limited larval swimming ability and their small size supports the hypothesis that the dispersal of species with planktonic larval stages is determined by the ocean currents and the pelagic larval duration (PLD)^83^. It is assumed that the maximum PLD is driven by type of larvae^13^. In our study we collected *Amphiophiura bullata* from a wide range of different habitats such as seamounts, abyssal plains and deep seafloor. This agrees with the wide distribution range known for this species that is documented from both the Indian and the Atlantic Oceans^84^. Their wide distribution may be reasoned by being a broadcasting species with lecithotrophic larvae^85^ which fosters a higher dispersal potential and therefore a wider species distribution especially when compared to brooding species^86^. As an example of known brooding species, our dataset contains *Ophiosemnotes clavigera* and *Ophiosabine anomala*^15,87^, and both species were only found at one site, highlighting the hypothesis that a brooding life cycle limits the dispersal potential of species. The variability in the reproductive strategies of brittle stars emphasizes the importance of considering the individual life cycle and lifestyle of the studied species when assessing biodiversity. Regionally distributed species are more vulnerable to threats than widespread species such as *Amphiophiura bullata.*

### Implications for the results of this paper

Assessing factors which shape species distribution remains challenging when we consider the large geographical distances between sampling locations and the relatively small size of the sampling area compared to the vast unsampled regions in the North Atlantic. Smaller scale variations, food availability and individual life traits of the species contribute to the composition of benthic communities, creating a mosaic of influences that vary across different spatial scales^60,65,75^. The interconnected complexity of biotic and abiotic factors contributing to biodiversity patterns cannot be attributed to one ultimate factor. The data presented herein offer an initial insight into species connectivity and distribution patterns in the North Atlantic Ocean while emphasizing the significance of expanding sampling points to facilitate a deeper understanding of connectivity in the deep sea. Despite the lack of a significant correlation between habitat heterogeneity and biodiversity, our observations suggest potential connections, particularly in structured environments such as cold-water coral reefs like Lóndsjúp, and it is evident that each sampled habitat exhibits a distinct species composition contributing to the overall biodiversity in the North Atlantic.

### Supplementary Information

Supplementary Information.

## Data Availability

The dataset generated for this study is available at the Barcode of Life Data System (BOLD, www.barcodinglife.org). The project is named “DS-OPHNA Ophiuroids from the North Atlantic” with the 10.5883/DS-OPHNA.
