# Supplementary material for: Unveiling ophiuroid biodiversity across North Atlantic habitats via an integrative perspective
Source: Sci Rep. 2024 Sep 2;14:20405. doi: 10.1038/s41598-024-71178-9 (PMC11369278; doi:10.1038/s41598-024-71178-9)
Supplement: Supplementary file 1 — Supplementary Information. [file 41598_2024_71178_MOESM1_ESM.pdf]

Supplementary Information

Table S1: Reference sequences used in this study. For each sequence the accession nb. Given in the NCBI database (www.ncbi.nlm.nih.gov) or the sample ID given in this study is documented as well as the sampling region of the specimen. The sequence from *Lytechinus variegatus* serves as the outgroup.

Species	Sampling Region	Accession Nb. /Sample ID	Reference
Ophiomitrella conferta (Koehler, 1922)	Australia	HQ946175.1	Biodiversity Institute of Ontario,
Ophiosabine cuspidata Lyman, 1878	Ireland	NUI2256A	Eichsteller et al., 2022
Ophiactis balli (W. Thompson, 1840)	North Sea	KX459004.1	Laakmann et al., 2018
Ophiopholis aculeata (Linnaeus, 1767)	USA, Washington	MZ580558.1	O'Mahoney et al., 2021
Ophiactis abyssicola (M. Sars, 1861)	New Zealand	KU895139	Hugall et al., 2016
Lytechinus variegatus (Lamarck, 1816)	Panama	MN683945	Collin et al., 2020

Table S2: *K2P genetic distance within all species. Calculated using 500 Bootstrap replications.*

**Species**	**Pairwise distance within the species (%)**
Ophiacantha bidentata	n/c
Ophiactis abyssicola	1,98
Ophiopus arcticus	1,54
Ophiotreta spectabilis	0,96
Ophiotholia sp.	n/c
Ophiura sarsii	3,08
Ophiura ljungmani	1,44
Ophiocten gracilis	0,59
Ophiosemnotes clavigera	0,08
Ophiacantha fraterna	n/c
Ophiacantha aculeata	n/c
Ophiactis balli	2,83
Ophiopholis aculeata	2,37
Ophiolycus purpureus	n/c
Amphiophiura bullata	0,69
cf. Ophiosphalma sp.	0,3
Ophiura robusta	0,27
Ophiuocten cf. umbraticum	0,88
Ophiosabine anomala	n/c
Ophiacantha simulans	n/c
cf. Ophiuroglypha sp. B	0,79
cf. Ophiuroglypha sp. A	0,64
Ophioplinthus sp.	n/c
Ophiosphalma armigerum	0,66
